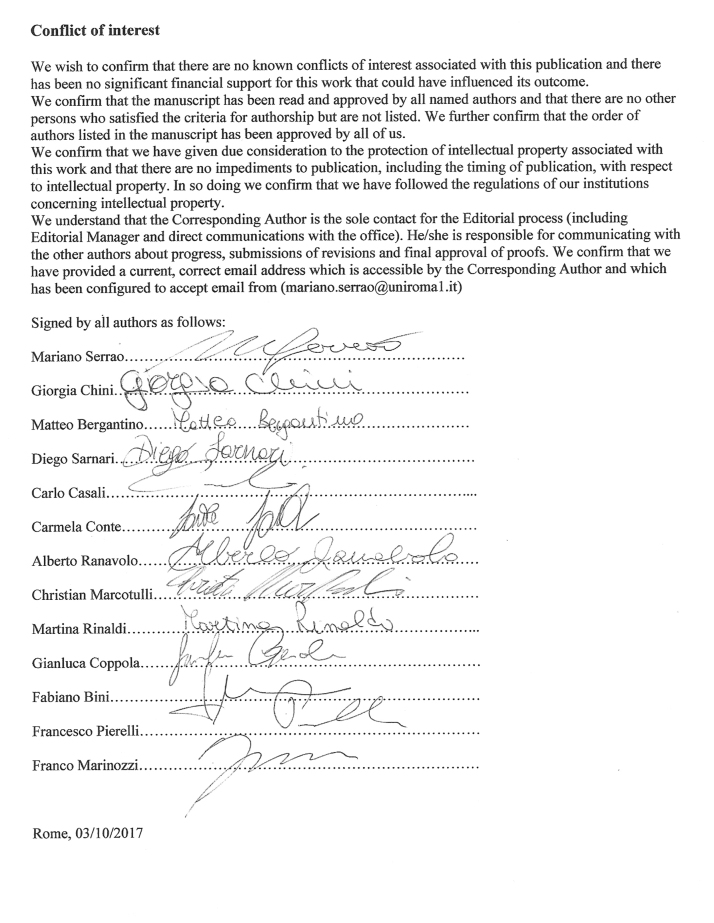# Dataset on gait patterns in degenerative neurological diseases

**DOI:** 10.1016/j.dib.2017.12.022

**Published:** 2017-12-12

**Authors:** Mariano Serrao, Giorgia Chini, Matteo Bergantino, Diego Sarnari, Carlo Casali, Carmela Conte, Alberto Ranavolo, Christian Marcotulli, Martina Rinaldi, Gianluca Coppola, Fabiano Bini, Francesco Pierelli, Franco Marinozzi

**Affiliations:** a Department of Medical and Surgical Sciences and Biotechnologies, Sapienza University of Rome, Corso della Repubblica 79, Latina 40100, Italy; b Movement Analysis LAB, Rehabilitation Centre Policlinico Italia, Piazza del Campidano 6, 00162 Rome, Italy; c Biolab3, Department of Engineering, Roma TRE University, Via Vito Volterra 62, 00149 Roma, Italy; d Department of Mechanical and Aerospace Engineering, “Sapienza” University of Rome, Via Eudossiana 18 – 00184 Roma, Italy; e Fondazione Don Gnocchi Foundation; Milan, Italy; f INAIL, Department of Occupational and Environmental Medicine, Epidemiology and Hygiene, Via Fontana Candida 1, 00040 Monte Porzio Catone, Italy; g G.B. Bietti Foundation-IRCCS, Department of Neurophysiology of Vision and Neurophthalmology, Via Livenza 3, 00198 Rome, Italy; h IRCCS Neuromed, Pozzilli (IS), Italy

## Abstract

We collected the gait parameters and lower limb joint kinematics of patients with three different types of primary degenerative neurological diseases: (i) cerebellar ataxia (19 patients), (ii) hereditary spastic paraparesis (26 patients), and (iii) Parkinson’s disease (32 patients). Sixty-five gender-age matched healthy subjects were enrolled as control group. An optoelectronic motion analysis system was used to measure time-distance parameters and lower limb joint kinematics during gait in both patients and healthy controls.

**Specifications Table**

**Table:** 

Subject area	*Biomechanics*
More specific subject area	*Movement disorders*
Type of data	*Tables*
How data was acquired	*The data were acquired using an optoelectronic motion analysis system (SMART-DX 500 System, BTS, Milan, Italy).*
Data format	*analyzed*
Experimental factors	*No pretreatment was performed on the sample*
Experimental features	*Subjects were asked to walk barefoot at a comfortable, self-selected speed along a walkway approximately 10 m in length while looking forward*
Data source location	*The data were recorded in the Motion Analysis Lab between October 2011 and May 2014 in Policlinico Italia, Rome, Italy*
Data accessibility	*The data are available with this article*

**Value of the data**

• The gait data provided here can be used for further analyses aimed at individuating uncommon subtypes of gait patterns.
• The data may serve as a benchmark for other researchers in the investigation on other types of gait disorders.
• Our data may be shared with those of other motion analysis LABs in order to improve the statistical analysis power.

## 1 Data

The data we are sharing with this article are gait spatio-temporal parameters and joint kinematics of patients affected by cerebellar ataxia, hereditary spastic paraplegia and Parkinson’s Disease. Sixty-five gender-age-speed matched healthy subjects were enrolled as control group [1]. Table 1, Table 2, Table 3, Table 4 shows patients’ and healthy controls’ anthropometric and clinical characteristics, while Table 5, Table 6, Table 7, Table 8 illustrates the patients’ and controls’ gait spatio-temporal parameters and joint kinematics.

**Table 1:** Cerebellar ataxic (CA) patients’ anthropometric and clinical characteristics.

**Subject-ID**	**Gender (F/M)**	**SARA-total**	**Disease duration (years)**	**Diagnosis**	**Age (years)**	**Weight (kg)**	**Height (m)**
CA -1	F	10	10	SAOA	60	52	1.5
CA -2	M	12.5	11	SAOA	58	70	1.69
CA -3	M	12	16	SAOA	46	69	1.67
CA -4	M	15	10	SCA1	45	69	1.74
CA -5	F	10	2	SAOA	64	63.5	1.62
CA -6	F	21	32	SCA1	32	54	1.59
CA -7	M	8	8	SCA1	43	66	1.74
CA -8	M	12	5	SCA2	35	73	1.73
CA -9	M	11	8	SCA1	49	72	1.76
CA -10	M	13.5	17	SAOA	47	91.5	1.7
CA -11	F	6	11	SAOA	71	54	1.58
CA -12	M	14	29	SAOA	46	72	1.71
CA -13	M	9	19	SAOA	49	75	1.73
CA -14	M	11	24	SCA3	54	76	1.7
CA -15	F	8	20	SCA3	50	64.5	1.66
CA -16	M	2	4	SCA1	33	51	1.63
CA -17	M	22	14	SCA2	40	87	1.67
CA -18	F	13	5	SCA2	48	56	1.53
CA -19	F	12.5	14	SAOA	54	68	1.48

**Table 2:** Hereditary spastic paraparesis (HSP) patients’ anthropometric and clinical characteristics.

**Subject-ID**	**Gender (F/M)**	**SPRS-total**	**Disease duration (years)**	**Diagnosis**	**Age (years)**	**Weight (kg)**	**Height (m)**
HSP-1	M	23	32	SPG4	66	63	1.62
HSP-2	M	7	12	SPG4	47	87	1.74
HSP-3	F	0	0	SPG4	30	78	1.52
HSP-4	F	28	33	SPG4	78	55.5	1.42
HSP-5	M	7	22	HSP-AD	35	75	1.84
HSP-6	M	17	15	SPG4	58	78	1.71
HSP-7	F	6	38	SPG4	43	57.5	1.62
HSP-8	M	12	3	Sporadic SPG	39	99	1.7
HSP-9	M	12	15	SPG4	28	81	1.81
HSP-10	M	25	32	SPG4	34	57	1.7
HSP-11	F	6	36	SPG4	66	57	1.54
HSP-12	M	12	11	SPG4	56	75	1.64
HSP-13	F	2	18	SPG4	21	58	1.63
HSP-14	M	35	25	HSP-AD	49	70	1.7
HSP-15	M	8	10	SPG4	24	104	1.77
HSP-16	M	24	13	SPG4	58	72.5	1.7
HSP-17	M	3	12	SPG-AD	25	85	1.8
HSP-18	F	4	5	SPG4	43	69	1.7
HSP-19	M	16	12	SPG4	49	109	1.82
HSP-20	F	31	32	SPG4	72	69	1.58
HSP-21	M	26	16	Sporadic SPG	30	75	1.72
HSP-22	M	28	29	SPG-AR	59	87	1.57
HSP-23	F	20	21	SPG-AR	56	73	1.59
HSP-24	F	18	21	SPG5	57	65.5	1.56
HSP-25	F	23	57	SPG31	72	77	1.49
HSP-26	F	12	48	SPG31	64	61	1.58

**Table 3:** Parkinson’s disease (PD) patients’ anthropometric and clinical characteristics.

**Subject-ID**	**Gender (F/M)**	**UPDRS III**	**Disease duration (years)**	**Diagnosis**	**Age (years)**	**Weight (kg)**	**Height (m)**
PD-1	M	15	11	IPD	60	66	1.61
PD-2	M	11	10	IPD	76	83.5	1.65
PD-3	F	6	4	IPD	66	58.5	1.55
PD-4	F	11	4	IPD	73	77	1.62
PD-5	F	26	4	IPD	76	69.5	1.54
PD-6	M	15	6	IPD	77	65	1.69
PD-7	M	12	3	IPD	75	85	1.65
PD-8	M	14	15	IPD	72	61	1.66
PD-9	M	17	9	IPD	70	71.5	1.7
PD-10	F	17	2	IPD	73	64.5	1.49
PD-11	F	21	2	IPD	70	62	1.5
PD-12	F	14	3	IPD	56	60	1.58
PD-13	M	19	6	IPD	73	59	1.54
PD-14	F	6	2	IPD	68	56.5	1.52
PD-15	M	18	14	IPD	61	70	1.53
PD-16	M	22	3	IPD	73	89	1.64
PD-17	F	28	5	IPD	70	93	1.55
PD-18	M	15	12	IPD	62	82.5	1.67
PD-19	F	17	9	IPD	73	77	1.58
PD-20	M	14	8	IPD	79	81	1.75
PD-21	M	10	7	IPD	66	90	1.69
PD-22	M	16	4	IPD	73	59	1.61
PD-23	M	11	4	IPD	74	80.5	1.69
PD-24	F	8	7	IPD	57	75	1.57
PD-25	F	20	6	IPD	72	61	1.55
PD-26	M	7	1	IPD	66	97	1.74
PD-27	M	13	6	IPD	71	88	1.66
PD-28	F	9	5	IPD	73	61.5	1.54
PD-29	M	14	9	IPD	50	82.5	1.74
PD-30	F	3	3	IPD	71	50	1.49
PD-31	M	11	3	IPD	73	63	1.58
PD-32	F	14	10	IPD	76	60	1.55

**Table 4:** Healthy controls’ anthropometric chacateristics.

**Subject-ID**	**Gender (F/M)**	**Age (years)**	**Weight (kg)**	**Height (m)**
HC-1	F	49	63	1.56
HC-2	M	71	92	1.83
HC-3	F	47	53.5	1.65
HC-4	F	59	63	1.56
HC-5	F	77	74	1.69
HC-6	M	38	74	1.75
HC-7	F	33	52	1.61
HC-8	F	38	50	1.62
HC-9	F	56	70	1.61
HC-10	F	43	56	1.58
HC-11	F	28	51	1.64
HC-12	M	46	76	1.73
HC-13	F	64	98	1.51
HC-14	F	43	52	1.6
HC-15	M	35	75	1.75
HC-16	M	57	88	1.79
HC-17	M	70	64.5	1.61
HC-18	F	74	76	1.7
HC-19	F	35	64	1.61
HC-20	F	53	55	1.58
HC-21	M	39	75	1.65
HC-22	F	54	67	1.52
HC-23	F	52	66	1.58
HC-24	M	56	83	1.8
HC-25	M	57	71	1.65
HC-26	M	57	71	1.69
HC-27	M	38	73	1.8
HC-28	F	44	49	1.65
HC-29	M	63	94	1.8
HC-30	F	38	56	1.64
HC-31	M	47	85.5	1.73
HC-32	F	60	63	1.61
HC-33	M	56	78	1.725
HC-34	F	73	73	1.54
HC-35	F	61	60	1.54
HC-36	M	63	75.5	1.77
HC-37	M	27	81	1.82
HC-38	M	75	74.5	1.74
HC-39	M	61	67	1.8
HC-40	F	46	64	1.61
HC-41	M	34	73	1.76
HC-42	M	49	95	1.81
HC-43	M	29	65	1.7
HC-44	M	46	80	1.69
HC-45	M	65	67.5	1.71
HC-46	F	26	64	1.53
HC-47	F	37	70	1.59
HC-48	F	35	66	1.64
HC-49	F	66	51.5	1.49
HC-50	M	43	94	1.74
HC-51	M	41	92	1.76
HC-52	F	45	64	1.66
HC-53	M	51	78	1.82
HC-54	F	41	72	1.72
HC-55	M	44	68	1.64
HC-56	F	54	51	1.64
HC-57	F	59	61	1.69
HC-58	M	54	94	1.81
HC-59	M	35	96	1.71
HC-60	M	45	83	1.82
HC-61	M	33	75	1.71
HC-62	F	73	51	1.49
HC-63	M	69	73	1.76
HC-64	F	45	56	1.75
HC-65	M	58	70.5	1.69

**Table 5:** Cerebellar ataxia **(**CA) patients’ gait spatio-temporal parameters and joint kinematics.

**Subject_ID**	**R Stance (%)**	**L Stance (%)**	**R Swing (%)**	**L Swing (%)**	**R Double Supp. (%)**	**L Double Supp.(%)**	**R Step Lenght (m)**	**L Step Lenght (m)**	**R Speed (m/s)**	**L Speed (m/s)**	**R Hip ROM (°)**	**L Hip ROM (°)**	**R Knee ROM (°)**	**L Knee ROM (°)**	**R Ankle ROM (°)**	**L Ankle ROM (°)**	**Cadence (n°step/min)**	**Step Width (m)**
CA -1	66.60	61.60	33.40	38.40	13.90	12.80	0.35	0.40	0.60	0.62	37.30	36.73	50.18	44.48	14.33	12.65	89.85	0.17
CA -2	64.60	63.90	35.40	36.10	13.20	15.50	0.48	0.46	0.76	0.77	46.20	35.62	65.12	58.02	19.68	22.30	89.88	0.19
CA -3	61.50	60.70	38.50	39.30	11.70	10.40	0.49	0.48	0.85	0.87	46.43	38.23	57.97	51.40	14.63	19.80	96.00	0.25
CA -4	63.20	59.60	36.80	40.40	12.80	9.80	0.62	0.57	1.03	1.03	53.20	54.00	60.87	61.27	19.40	15.53	95.00	0.22
CA -5	64.40	65.60	35.60	34.40	15.80	14.30	0.38	0.40	0.77	0.74	26.90	37.34	49.00	48.54	20.66	19.36	110.64	0.16
CA -6	64.20	67.80	35.80	32.20	17.60	13.10	0.24	0.38	0.33	0.36	41.63	47.40	54.87	50.80	15.90	15.47	62.60	0.15
CA -7	61.10	61.70	38.90	38.30	10.30	10.10	0.55	0.55	1.20	1.16	42.67	40.93	55.23	57.43	13.30	17.10	117.40	0.24
CA -8	62.70	63.90	37.30	36.10	13.90	13.20	0.49	0.42	0.81	0.82	37.88	41.64	50.90	59.54	16.24	14.86	99.20	0.24
CA -9	64.40	61.30	35.40	38.70	14.00	13.10	0.41	0.45	0.77	0.79	41.98	37.70	53.23	38.70	12.18	9.13	99.00	0.20
CA -10	72.30	75.20	27.70	24.80	21.20	25.60	0.32	0.33	0.41	0.44	29.70	38.72	44.66	43.58	19.14	11.28	73.20	0.26
CA -11	64.10	64.50	35.90	35.50	14.00	14.60	0.43	0.38	0.52	0.54	32.10	39.78	47.18	56.00	17.68	19.95	73.50	0.24
CA -12	59.80	60.90	40.20	39.10	13.20	8.20	0.54	0.44	1.05	0.92	36.96	41.40	54.34	50.22	16.40	14.10	110.80	0.24
CA -13	55.80	56.10	44.20	43.90	6.50	4.90	0.64	0.66	1.31	1.31	43.92	43.48	62.20	65.28	28.06	27.20	105.60	0.17
CA -14	61.20	60.90	38.60	39.10	13.50	11.10	0.48	0.50	0.86	0.87	40.90	36.16	52.34	53.58	18.66	15.26	97.44	0.19
CA -15	61.80	66.90	38.20	33.10	14.40	13.50	0.44	0.44	0.68	0.71	33.42	43.14	45.94	62.07	21.80	23.62	89.40	0.21
CA -16	60.70	60.00	39.30	40.00	10.20	10.30	0.57	0.55	1.13	1.14	49.73	51.78	68.08	70.03	20.80	22.90	110.50	0.16
CA -17	69.30	72.10	30.70	27.90	21.20	21.20	0.30	0.28	0.49	0.49	37.16	38.24	37.22	63.14	11.24	8.10	93.80	0.27
CA -18	64.00	60.60	36.00	39.40	12.50	11.30	0.41	0.43	0.79	0.77	40.34	41.66	59.90	62.22	17.36	13.70	102.84	0.17
CA -19	64.10	67.20	35.90	32.80	16.00	15.30	0.32	0.33	0.64	0.64	44.50	41.68	56.26	50.92	10.96	12.54	105.70	0.23

**Table 6:** Hereditary spastica paraparesis (HSP) patients’ gait spatio-temporal parameters and joint kinematics.

**Subject-ID**	**R Stance (%)**	**L Stance (%)**	**R Swing (%)**	**L Swing (%)**	**R Double Supp. (%)**	**L Double Supp.(%)**	**R Step Lenght (m)**	**L Step Lenght (m)**	**R Speed (m/s)**	**L Speed (m/s)**	**R Hip ROM (°)**	**L Hip ROM (°)**	**R Knee ROM (°)**	**L Knee ROM (°)**	**R Ankle ROM (°)**	**L Ankle ROM (°)**	**Cadence (n°step/min)**	**Step Width (m)**
HSP-1	75.10	78.80	24.40	21.20	30.40	22.20	0.30	0.33	0.10	0.10	45.90	33.40	59.70	53.20	24.80	22.70	18.00	0.11
HSP-2	62.90	66.40	37.10	33.60	13.70	15.40	0.50	0.45	0.75	0.76	43.40	38.30	52.47	52.80	22.40	21.20	90.20	0.21
HSP-3	62.50	64.00	37.50	36.00	12.80	14.20	0.50	0.49	0.81	0.82	41.80	35.90	56.10	58.40	31.10	45.80	93.20	0.14
HSP-4	84.00	85.00	16.00	15.00	33.80	33.20	0.13	0.25	0.15	0.15	18.00	13.85	28.35	33.68	21.98	24.93	44.10	0.20
HSP-5	59.80	60.70	40.30	39.30	9.90	11.60	0.58	0.64	1.17	1.21	44.40	48.35	58.75	68.73	29.05	29.20	110.60	0.17
HSP-6	78.80	79.10	21.20	20.90	28.10	28.30	0.22	0.30	0.15	0.15	44.40	33.60	57.00	39.10	23.60	11.50	33.15	0.18
HSP-7	61.10	60.50	38.90	39.50	10.50	11.60	0.49	0.45	0.83	0.84	42.38	41.83	39.40	44.80	19.78	17.88	98.55	0.14
HSP-8	61.40	59.40	38.60	40.60	10.70	9.60	0.55	0.55	1.00	0.99	48.70	40.22	46.04	47.26	15.68	14.76	98.40	0.18
HSP-9	63.00	63.20	37.00	36.80	11.30	14.20	0.55	0.58	0.89	0.89	36.20	38.96	36.14	41.32	23.96	19.94	89.16	0.19
HSP-10	64.50	72.50	35.50	27.50	20.10	18.30	0.25	0.33	0.33	0.34	20.60	39.30	24.60	47.40	12.70	16.90	66.80	0.19
HSP-11	63.20	62.50	36.80	37.50	13.80	12.70	0.47	0.48	0.79	0.82	45.70	44.10	59.00	62.50	34.90	27.60	95.60	0.13
HSP-12	62.90	63.70	37.10	36.30	14.50	12.00	0.45	0.46	0.71	0.72	39.70	43.10	46.50	48.50	19.80	20.60	88.20	0.17
HSP-13	58.90	59.30	41.10	40.70	8.60	8.80	0.56	0.57	1.07	1.09	46.70	49.17	62.73	61.43	37.27	28.97	106.60	0.15
HSP-14	77.30	80.20	22.70	19.80	27.40	32.90	0.30	0.24	0.14	0.15	32.38	40.85	39.15	47.28	25.53	31.30	29.40	0.14
HSP-15	68.60	66.50	31.40	33.50	15.80	20.50	0.38	0.32	0.42	0.43	31.80	29.40	38.23	33.20	18.90	21.53	68.20	0.14
HSP-16	60.80	59.60	39.20	40.40	12.60	8.20	0.60	0.63	1.12	1.12	44.77	48.83	44.17	44.03	23.30	28.37	101.80	0.21
HSP-17	66.60	65.60	32.80	34.40	13.90	17.60	0.40	0.45	0.63	0.63	31.20	33.83	36.53	36.07	15.03	13.03	85.20	0.17
HSP-18	60.10	60.40	39.90	39.60	10.50	10.10	0.61	0.58	1.13	1.14	50.73	43.50	63.63	57.05	37.85	27.73	106.00	0.14
HSP-19	61.20	61.60	38.80	38.40	12.20	11.10	0.62	0.60	1.18	1.19	45.76	45.80	27.96	40.52	18.58	23.30	107.76	0.14
HSP-20	88.30	86.40	11.70	13.60	37.90	35.70	0.13	0.15	0.12	0.12	25.55	16.20	37.35	28.55	13.95	18.68	51.00	0.24
HSP-21	78.60	82.60	21.40	17.40	28.40	32.30	0.17	0.17	0.14	0.13	11.72	10.28	17.18	16.88	26.60	29.32	46.08	0.28
HSP-22	66.50	70.50	33.50	29.50	16.90	20.30	0.31	0.35	0.46	0.46	40.06	37.66	36.26	25.66	12.84	22.80	81.12	0.17
HSP-23	79.30	80.80	20.70	19.20	29.00	31.00	0.26	0.25	0.19	0.18	28.70	45.78	31.40	47.63	8.33	10.45	41.55	0.13
HSP-24	68.10	65.90	31.90	34.10	18.10	15.70	0.37	0.34	0.63	0.61	39.87	32.13	45.97	35.23	23.87	21.00	94.60	0.16
HSP-25	72.80	71.00	27.20	29.00	20.20	21.90	0.34	0.39	0.47	0.47	35.58	41.92	34.44	37.26	29.58	21.74	70.68	0.16
HSP-26	60.30	57.60	39.70	42.40	8.60	9.20	0.46	0.45	1.02	1.02	32.80	42.72	46.84	41.10	28.78	22.90	123.60	0.17

**Table 7:** Parkinson’s disease (PD) patients’ gait spatio-temporal parameters and joint kinematics.

**Subject_ID**	**R Stance (%)**	**L Stance (%)**	**R Swing (%)**	**L Swing (%)**	**R Double Supp. (%)**	**L Double Supp.(%)**	**R Step Lenght (m)**	**L Step Lenght (m)**	**R Speed (m/s)**	**L Speed (m/s)**	**R Hip ROM (°)**	**L Hip ROM (°)**	**R Knee ROM (°)**	**L Knee ROM (°)**	**R Ankle ROM (°)**	**L Ankle ROM (°)**	**Cadence (n°step/min)**	**Step Width (m)**
PD-1	58.90	63.70	41.10	36.30	12.80	9.80	0.44	0.56	0.82	0.83	32.47	44.47	35.13	57.33	23.63	27.80	92.80	0.15
PD-2	68.10	67.30	31.90	32.70	19.70	15.60	0.29	0.23	0.44	0.45	19.12	20.66	29.72	31.02	17.48	16.70	99.24	0.15
PD-3	60.80	62.20	39.20	37.80	12.30	11.90	0.45	0.46	0.92	0.93	41.60	40.63	52.10	49.85	22.73	22.55	110.55	0.16
PD-4	63.40	62.00	36.60	38.00	12.20	12.10	0.52	0.55	0.89	0.91	38.90	36.18	57.75	57.90	29.65	34.55	93.00	0.15
PD-5	69.50	72.40	30.50	27.60	16.30	24.30	0.34	0.31	0.41	0.40	28.10	26.68	42.55	41.23	26.63	24.85	69.45	0.18
PD-6	61.40	57.90	38.60	42.10	8.90	10.90	0.44	0.46	0.96	0.98	35.53	31.53	55.33	49.93	19.33	18.65	116.20	0.15
PD-7	61.70	60.90	38.30	39.10	11.50	11.70	0.52	0.50	0.84	0.87	40.60	43.70	49.97	50.77	20.30	22.30	93.80	0.17
PD-8	57.90	58.70	42.10	41.30	8.30	7.40	0.56	0.55	0.96	0.95	42.30	38.50	61.10	56.90	27.33	23.10	94.40	0.17
PD-9	60.70	59.00	39.30	41.00	10.10	9.30	0.51	0.47	0.92	0.92	38.05	33.38	53.08	40.98	21.10	16.65	103.95	0.15
PD-10	61.00	60.70	39.00	39.30	12.60	9.20	0.38	0.38	0.70	0.70	31.66	29.64	46.40	43.97	20.97	22.23	102.48	0.15
PD-11	77.70	75.30	22.30	24.70	29.60	24.30	0.14	0.23	0.19	0.19	25.82	21.50	27.50	26.62	11.84	11.92	59.40	0.16
PD-12	66.40	65.10	33.60	34.90	14.40	17.50	0.40	0.39	0.59	0.65	35.00	33.27	41.23	40.00	19.03	21.43	89.00	0.14
PD-13	63.80	64.30	36.20	35.70	15.30	13.30	0.34	0.36	0.52	0.52	30.23	30.53	50.25	57.80	21.28	24.50	83.70	0.16
PD-14	60.40	59.00	39.60	41.00	10.30	9.80	0.46	0.48	0.90	0.90	44.83	41.50	52.77	45.60	20.60	23.10	105.40	0.14
PD-15	63.10	62.50	36.90	37.50	13.10	12.80	0.37	0.43	0.96	0.97	36.30	38.18	50.15	52.25	19.55	20.35	133.65	0.18
PD-16	64.20	64.30	35.80	35.70	12.60	17.30	0.43	0.44	0.73	0.74	32.77	29.97	46.67	43.83	25.97	23.73	94.60	0.15
PD-17	70.00	64.80	30.00	35.20	19.60	15.60	0.31	0.37	0.51	0.50	29.40	28.90	42.75	32.85	22.48	27.05	86.85	0.16
PD-18	66.10	63.00	33.90	37.00	14.60	14.80	0.36	0.37	0.66	0.66	29.34	25.62	48.70	37.66	18.00	13.32	100.80	0.16
PD-19	62.70	64.50	37.30	1.60	14.60	13.00	0.38	0.35	0.66	0.66	35.33	29.93	50.23	39.83	17.65	18.13	100.50	0.13
PD-20	59.20	60.60	40.80	39.40	9.50	10.10	0.40	0.40	0.84	0.82	29.85	28.10	44.50	47.48	15.22	20.48	116.40	0.17
PD-21	61.40	61.90	38.60	38.10	11.80	11.30	0.47	0.41	0.95	0.97	27.20	30.96	45.76	55.22	18.96	28.64	122.40	0.19
PD-22	61.40	62.50	38.60	37.50	10.90	13.10	0.35	0.37	0.70	0.71	29.04	29.00	37.14	39.34	17.52	19.78	111.00	0.14
PD-23	60.80	60.20	39.20	39.80	11.10	10.00	0.57	0.57	1.19	1.22	44.25	45.61	56.58	74.15	26.10	27.08	118.20	0.12
PD-24	55.50	64.80	44.50	35.20	11.40	9.60	0.52	0.52	0.81	0.80	45.33	47.30	45.83	52.95	23.38	32.30	84.75	0.15
PD-25	65.40	62.70	34.60	37.30	14.50	13.40	0.43	0.46	0.88	0.90	39.53	37.83	52.30	54.50	27.50	24.60	110.00	0.15
PD-26	63.70	59.90	36.30	40.10	12.00	11.40	0.49	0.49	0.98	1.00	35.53	36.98	54.58	61.20	29.05	26.08	112.20	0.18
PD-27	62.10	62.20	37.90	37.80	11.30	13.40	0.44	0.51	0.95	0.95	44.03	38.33	53.83	52.23	19.38	18.38	112.80	0.16
PD-28	68.50	66.30	31.50	33.70	16.40	18.20	0.35	0.34	0.42	0.42	26.98	29.84	43.14	44.60	29.76	25.40	67.92	0.17
PD-29	61.30	59.30	38.70	40.70	11.20	9.00	0.58	0.59	1.18	1.19	19.96	20.22	56.27	48.00	24.87	23.83	112.80	0.14
PD-30	61.70	63.00	38.30	37.00	11.80	11.90	0.40	0.42	0.78	0.78	38.37	40.20	55.30	55.37	17.03	23.57	107.60	0.15
PD-31	64.50	64.40	35.50	35.60	14.30	14.50	0.41	0.41	0.77	0.78	27.43	29.27	45.53	45.03	22.47	16.67	105.60	0.19
PD-32	71.20	70.60	28.80	29.40	22.10	21.50	0.12	0.12	0.26	0.25	15.30	15.87	23.63	27.23	14.00	16.47	82.40	0.18

**Table 8:** Healthy controls’ (HC) gait spatio-temporal parameters and joint kinematics.

**Subject_ID**	**R Stance (%)**	**L Stance (%)**	**R Swing (%)**	**L Swing (%)**	**R Double Supp. (%)**	**L Double Supp.(%)**	**R Step Lenght (m)**	**L Step Lenght (m)**	**R Speed (m/s)**	**L Speed (m/s)**	**R Hip ROM (°)**	**L Hip ROM (°)**	**R Knee ROM (°)**	**L Knee ROM (°)**	**R Ankle ROM (°)**	**L Ankle ROM (°)**	**Cadence (n°step/min)**	**Step Width (m)**
HC-1	63.40	62.00	36.60	38.00	12.70	12.40	0.48	0.48	0.82	0.82	39.50	38.60	59.70	68.00	35.30	26.33	92.40	0.14
HC-2	63.40	64.70	36.60	35.30	13.60	13.90	0.56	0.55	0.86	0.87	43.78	36.70	58.63	57.45	26.73	36.70	87.45	0.19
HC-3	66.70	67.70	33.30	32.30	18.00	16.30	0.44	0.43	0.41	0.42	41.02	35.00	51.56	56.72	39.36	28.72	54.60	0.16
HC-4	64.50	64.00	35.50	36.00	14.80	14.30	0.46	0.45	0.62	0.61	38.14	36.72	60.78	54.24	35.98	29.88	75.60	0.15
HC-5	66.60	66.30	33.40	33.70	15.40	17.70	0.38	0.47	0.64	0.65	36.08	34.90	47.04	49.76	21.56	34.90	85.44	0.14
HC-6	64.30	65.10	35.70	34.90	14.60	14.40	0.47	0.44	0.60	0.59	36.34	36.10	58.60	58.92	20.18	22.54	73.44	0.20
HC-7	64.10	61.50	35.90	38.50	13.50	13.60	0.50	0.53	0.76	0.78	41.78	43.83	61.90	58.63	27.43	43.83	82.35	0.15
HC-8	66.70	64.30	33.30	35.70	15.70	15.40	0.45	0.44	0.63	0.63	35.32	38.32	59.08	60.50	27.18	38.32	80.52	0.16
HC-9	64.60	64.10	35.40	35.90	14.20	15.60	0.46	0.46	0.78	0.79	35.57	41.63	55.93	59.87	27.10	30.50	95.60	0.13
HC-10	60.90	63.50	39.10	36.50	10.70	15.10	0.51	0.52	0.79	0.81	44.20	45.73	64.33	64.17	24.83	26.33	86.20	0.15
HC-11	61.30	62.70	38.70	37.30	12.80	11.30	0.48	0.49	0.82	0.80	40.17	40.53	60.83	62.20	24.77	40.53	91.80	0.13
HC-12	64.90	63.50	35.10	36.50	13.70	15.20	0.44	0.44	0.72	0.72	35.66	36.14	56.66	54.68	21.84	22.10	90.48	0.19
HC-13	66.90	66.20	33.10	33.80	14.80	16.80	0.50	0.43	0.83	0.86	37.80	41.15	51.50	57.65	31.25	41.15	101.10	0.16
HC-14	63.30	62.40	36.70	37.60	13.20	12.10	0.48	0.46	0.72	0.72	44.70	44.22	65.32	72.78	35.00	29.38	86.04	0.16
HC-15	65.40	64.80	34.60	35.20	14.30	15.30	0.45	0.48	0.68	0.68	33.57	32.63	52.90	52.53	26.30	32.63	81.20	0.17
HC-16	64.70	62.00	35.30	38.00	13.10	14.10	0.54	0.56	0.83	0.86	41.26	45.66	56.06	54.40	29.24	34.96	85.08	0.17
HC-17	65.80	64.70	34.20	35.30	16.40	15.80	0.43	0.42	0.54	0.55	37.52	39.08	39.00	43.90	17.38	39.08	72.24	0.12
HC-18	65.00	65.40	35.00	34.60	15.60	15.10	0.42	0.47	0.68	0.68	35.16	33.98	42.94	50.10	24.88	33.98	84.12	0.14
HC-19	62.00	61.80	38.00	38.20	11.50	13.40	0.57	0.55	0.98	0.98	49.00	47.87	57.80	56.30	37.37	47.87	95.40	0.14
HC-20	60.80	61.70	39.20	38.30	12.00	10.90	0.49	0.50	0.97	0.97	47.60	46.73	67.40	57.77	25.27	33.87	106.80	0.13
HC-21	62.50	63.00	37.50	37.00	13.00	12.40	0.50	0.46	0.77	0.77	36.68	36.70	51.60	51.56	21.70	21.42	88.92	0.15
HC-22	66.60	67.40	33.40	32.60	18.20	15.60	0.44	0.44	0.59	0.59	36.68	37.96	57.46	56.36	29.82	31.48	73.56	0.13
HC-23	62.90	62.70	37.10	37.30	13.40	12.00	0.47	0.42	0.79	0.79	30.82	34.36	49.50	48.32	24.20	28.46	98.04	0.13
HC-24	62.70	61.90	37.30	38.10	11.30	12.50	0.47	0.49	0.85	0.87	36.63	35.20	58.88	59.50	25.65	20.85	98.85	0.16
HC-25	64.40	64.80	35.60	35.20	12.60	16.10	0.46	0.53	0.73	0.75	36.00	34.50	54.35	52.15	30.30	34.50	82.50	0.19
HC-26	63.80	64.30	36.20	35.70	14.50	13.60	0.44	0.48	0.66	0.66	34.90	41.23	47.55	56.80	27.08	28.10	78.00	0.16
HC-27	59.00	59.30	41.00	40.70	9.30	9.30	0.70	0.63	1.23	1.20	41.43	42.43	67.37	71.57	40.43	42.43	104.40	0.21
HC-28	63.30	62.00	36.70	38.00	12.50	13.40	0.47	0.47	0.73	0.72	36.23	37.57	52.67	57.00	27.50	37.57	85.60	0.13
HC-29	66.40	63.80	33.60	36.20	15.50	15.30	0.42	0.46	0.59	0.61	35.62	37.82	52.86	56.74	20.82	19.28	73.92	0.19
HC-30	63.10	62.10	36.90	37.90	14.00	12.30	0.45	0.44	0.74	0.73	34.62	36.78	58.26	63.08	29.70	36.78	90.60	0.72
HC-31	66.80	64.10	33.20	35.90	16.80	15.00	0.52	0.54	0.84	0.86	43.32	47.10	53.58	59.70	27.20	32.18	88.80	0.21
HC-32	63.80	64.20	36.20	35.80	15.00	13.20	0.52	0.49	0.76	0.76	40.24	42.30	60.26	60.36	27.08	42.30	83.76	0.13
HC-33	64.40	64.10	35.60	35.90	14.20	13.60	0.45	0.46	0.70	0.69	39.38	33.58	44.83	47.93	22.60	21.25	85.35	0.17
HC-34	63.80	63.00	36.20	37.00	13.60	13.00	0.44	0.42	0.71	0.74	33.62	35.86	47.22	50.10	20.06	35.86	95.52	0.16
HC-35	65.00	62.30	35.00	37.70	14.50	13.20	0.49	0.51	0.81	0.80	41.83	41.40	58.35	62.48	31.68	41.40	90.00	0.14
HC-36	63.30	62.30	36.70	37.70	13.30	12.50	0.60	0.56	0.92	0.93	43.08	44.58	52.98	47.74	23.60	44.58	88.92	0.17
HC-37	67.40	66.10	32.60	33.90	18.30	18.00	0.41	0.34	0.52	0.53	28.80	26.24	53.30	45.24	18.40	26.24	74.64	0.20
HC-38	62.70	64.40	37.30	35.60	14.10	14.00	0.49	0.49	0.72	0.70	39.42	41.76	47.18	45.06	26.54	41.76	82.32	0.20
HC-39	60.70	60.50	39.30	39.50	11.40	9.70	0.61	0.58	1.02	1.01	39.90	42.33	49.57	58.23	23.50	42.33	93.40	0.15
HC-40	61.00	60.90	39.00	39.10	11.40	10.90	0.55	0.51	0.86	0.87	40.86	46.36	60.26	52.70	33.40	35.12	89.88	0.13
HC-41	60.60	61.10	39.40	38.90	9.80	12.10	0.60	0.63	1.06	1.05	48.25	44.75	60.50	60.33	37.50	44.75	94.50	0.16
HC-42	64.90	65.30	35.10	34.70	14.60	15.90	0.49	0.46	0.66	0.67	35.50	37.90	44.95	46.75	18.05	18.00	77.40	0.19
HC-43	62.00	60.50	38.00	39.50	11.10	11.90	0.54	0.54	1.09	1.10	36.18	39.13	50.48	53.83	25.30	39.13	110.40	0.15
HC-44	67.50	66.30	32.50	33.70	17.20	16.20	0.40	0.43	0.63	0.62	33.55	35.00	54.50	51.65	28.70	35.00	84.30	0.20
HC-45	68.30	67.80	31.70	32.20	18.60	17.60	0.43	0.47	0.57	0.57	37.87	39.43	56.87	54.40	28.47	39.43	70.00	0.15
HC-46	65.50	66.50	34.50	33.50	17.00	15.40	0.43	0.44	0.58	0.59	40.13	38.63	56.43	59.43	23.73	38.63	75.45	0.15
HC-47	63.20	61.70	36.80	38.30	13.00	11.90	0.48	0.46	0.93	0.92	36.80	38.75	55.35	55.60	26.40	38.75	109.80	0.20
HC-48	66.10	64.90	33.90	35.10	15.40	15.60	0.46	0.46	0.70	0.71	35.58	36.34	45.18	57.24	30.82	36.34	87.60	0.15
HC-49	61.60	61.20	38.40	38.80	11.60	10.90	0.49	0.47	0.91	0.91	48.88	47.68	58.15	66.40	27.78	47.68	104.40	0.12
HC-50	67.40	67.50	32.60	32.50	17.40	17.30	0.35	0.42	0.55	0.56	26.04	28.94	34.06	43.62	13.94	16.30	81.12	0.22
HC-51	60.90	61.00	39.10	39.00	11.20	11.50	0.60	0.56	0.88	0.87	36.12	37.38	60.34	60.68	34.16	30.20	84.00	0.20
HC-52	61.20	60.60	38.80	39.00	11.50	11.60	0.56	0.52	0.78	0.76	48.34	43.66	61.08	62.34	30.82	34.20	79.56	0.15
HC-53	60.30	60.90	39.70	39.10	9.70	11.20	0.60	0.64	1.20	1.22	43.67	45.53	45.77	52.10	31.27	45.53	102.20	0.18
HC-54	66.10	64.90	33.90	35.10	15.10	15.80	0.43	0.43	0.60	0.59	34.04	30.88	48.10	49.60	18.02	30.88	75.36	0.16
HC-55	64.70	65.70	35.30	34.30	15.90	14.10	0.43	0.48	0.60	0.59	38.18	35.66	51.16	48.62	20.00	27.60	97.20	0.16
HC-56	59.70	59.90	40.30	40.10	12.20	10.00	0.51	0.49	0.89	0.89	39.70	40.63	60.78	65.35	26.25	40.63	99.30	0.14
HC-57	65.40	67.60	34.60	32.40	16.70	16.40	0.46	0.46	0.57	0.57	41.45	43.10	57.80	59.35	39.00	43.10	68.10	0.12
HC-58	63.70	63.80	36.30	36.10	13.70	13.50	0.54	0.50	0.84	0.84	41.94	43.36	58.26	55.16	19.88	21.08	90.36	0.21
HC-59	65.20	64.20	34.80	35.80	15.30	14.10	0.38	0.41	0.70	0.70	29.88	30.84	49.44	48.60	19.66	30.84	97.44	0.21
HC-60	63.80	63.90	36.20	36.10	12.70	14.20	0.54	0.50	0.70	0.70	42.38	42.86	59.94	59.68	24.88	23.22	95.76	0.19
HC-61	64.30	63.30	35.70	36.70	14.10	13.50	0.48	0.54	0.88	0.88	41.07	41.57	55.03	59.30	26.00	41.57	94.20	0.16
HC-62	58.80	60.50	41.20	39.50	10.50	7.30	0.49	0.45	0.91	0.89	41.23	41.20	53.17	60.93	33.17	41.20	103.20	0.14
HC-63	65.00	65.80	35.00	34.20	16.10	14.50	0.44	0.48	0.57	0.57	36.08	38.56	40.44	47.44	16.48	38.56	69.72	0.15
HC-64	64.10	63.60	35.90	36.40	14.40	13.10	0.48	0.52	0.73	0.74	37.78	36.62	55.86	50.32	34.88	36.62	82.08	0.15
HC-65	65.80	63.60	34.20	36.40	14.50	14.40	0.42	0.43	0.71	0.70	33.38	33.18	50.44	49.18	23.26	33.18	92.64	0.16

## 2 Experimental design, materials and methods

Patients were asked to walk barefoot at a comfortable, self-selected speed along a walkway approximately 10 m in length while looking forward, while controls were asked to walk at slow speed. At least five trials were recorded for each patient and for each control. To avoid muscle fatigue, each trial was separated by a one-minute rest period.

Three-dimensional (3-D) marker trajectories were recorded using a frame-by-frame acquisition system (SMART Capture - BTS, Milan, Italy) and labeled using a frame-by-frame tracking system (SMART Tracker - BTS, Milan, Italy). Marker position data were interpolated and low-pass filtered using a zero-lag fourth-order Butterworth filter (6 Hz), [2] and analyzed using 3-D reconstruction software (SMART Analyzer, BTS, Milan, Italy) and MATLAB software (MATLAB 7.4.0, MathWorks, Natick, MA, USA).

The following time parameters were calculated for each subject: stance duration (time interval between two consecutive heel strikes of the same lower limb), swing duration (time interval between toe off and the next heel strike of the same lower limb), and double support duration (time interval with both feet on the floor), all expressed as a percentage of the stride duration. The following spatial parameters were computed for all the enrolled subjects: step length and step width, both expressed in meters. Furthermore, walking speed (m/s) and cadence (steps/min) were calculated for each subject.

The anatomical angles were computed for hip, knee, and ankle joints in the sagittal plane. From these variables, the RoM at each joint or segment, defined as the difference between the maximum and minimum value during the gait cycles, were derived. For each subject and each trial, kinematic data between two consecutive heel strikes of the same limb were time-normalized to 101 points, i.e. 0–100 % of the gait cycle, using a polynomial procedure, in order to exclude the influence of different stride durations, [3].

## Figures and Tables

**Fig. S1:**